# Addressing Ethical, Social, and Cultural Issues in Global Health Research

**DOI:** 10.1371/journal.pntd.0002227

**Published:** 2013-08-08

**Authors:** James V. Lavery, Shane K. Green, Sunita V. S. Bandewar, Anant Bhan, Abdallah Daar, Claudia I. Emerson, Hassan Masum, Filippo M. Randazzo, Jerome A. Singh, Ross E. G. Upshur, Peter A. Singer

**Affiliations:** 1 Ethical, Social and Cultural Program for Global Health, Centre for Research on Inner City Health and Centre for Global Health Research, Keenan Research Centre in the Li Ka Shing Knowledge Institute of St. Michael's Hospital, Toronto, Ontario, Canada; 2 Dalla Lana School of Public Health and Joint Centre for Bioethics, University of Toronto, Toronto, Ontario, Canada; 3 Ethical, Social and Cultural Program for Global Health, Sandra Rotman Centre, University Health Network & University of Toronto, Toronto, Ontario, Canada; 4 Bill & Melinda Gates Foundation, Seattle, Washington, United States of America; 5 Centre for the AIDS Programme of Research in South Africa (CAPRISA), Doris Duke Medical Research Institute, Nelson R Mandela School of Medicine, University of KwaZulu-Natal, Congella, Durban, South Africa; 6 Department of Family and Community Medicine, University of Toronto, Toronto, Ontario, Canada; The George Washington University Medical Center, United States of America

**Summary**

The purpose of this paper is to encourage reflection among the global health research community and the research ethics community about how a wide range of ethical, social, and cultural (ESC) influences on the conduct, success, and impact of global health research can best be addressed by consultation services in research ethics (CSRE). We draw on lessons we have learned during our experiences with the ESC Program of the Grand Challenges in Global Health initiative to propose key features of CSRE that may prove useful for those designing or implementing similar programs.

## Introduction

The past decade has seen unparalleled investment in large-scale global health science initiatives and international research consortia, such as the International HapMap Project, Grand Challenges in Global Health program, and International AIDS Vaccine Initiative. These initiatives have resulted in promising advances, such as candidate vaccines for malaria and HIV, nutritionally enhanced staple crops, novel vector control strategies, and an advanced understanding of human genetic diversity. They have also reflected the growing emphasis on innovation in global health and on the urgent need to test innovations in real-world settings, especially resource-constrained ones, to determine their potential effectiveness and value. Alongside—and necessitated by—these shifts in global health research, there has also been a broadening in the conversation about the ethical aspects of that research, from an almost singular focus on standard of care issues [1] to a more holistic consideration of a wide range of ethical, social, and cultural (ESC) influences on the conduct, success, and impact of biomedical science on underlying public health problems.

This broadening, in turn, has helped to fuel a growing interest within the health research community in consultation services in research ethics (CSRE). These are teams of experts in research ethics, typically based at academic bioethics centres, that provide advice and guidance to researchers and institutions about ethical issues that arise in the design and conduct of research. Since first proposed [2], there has been some attention to the evolution of CSREs in the literature [3], [4], with much of the focus on how to achieve an appropriate balance between the advisory/consulting role of the emerging CSREs and the review, monitoring, and oversight responsibilities of their counterpart institutional review boards (IRBs) [5]. Most recently, an article published in *Science Translational Medicine* from the Stanford University CSRE has provided important insights into circumstances that the authors argue should “trigger" investigators to seek consultations with the service [4]. Although the authors point to “research in developing nations" as one such trigger, there continues to be a gap in the literature about why and how CSREs might play an important role in proactively considering and helping to address the unique ESC challenges posed by global health research—in particular, research in low- and middle-income countries (LMICs) that is funded and conducted, in whole or in part, by organizations and investigators from high-income countries—and thereby provide a valuable complement to customary institutional research ethics review for this type of research.

The purpose of this paper is to encourage reflection among the global health research community, including funders, researchers, research institutions, and administrators of large-scale global health research initiatives, about how ESC issues can best be addressed within these initiatives. We draw on lessons we have learned during our experiences with the Ethical, Social and Cultural Program (ESC Program) of the Grand Challenges in Global Health (GCGH) initiative, funded by the Bill & Melinda Gates Foundation between 2005–2011 [6], to propose key features of a focused CSRE, which may prove useful for those designing or implementing similar programs.

## Key Features of an ESC Program for Global Health Research

### 1. Integrate ESC Consultation with the Planning and Performance of the Research

Integrating science and ethics is fundamentally about acknowledging that values permeate not only trials and applications of new technologies, but *all* aspects along the “critical path" of the scientific process—from discovery science through development of novel products and technologies to their effective delivery to end users, e.g., patients, consumers, communities, public health authorities—and identifying where lack of sufficient attention to ESC issues can undermine the ethics, social value, quality, feasibility, or sustainability of the science and its outputs. Effective integration enables proactive, deeply informed, interdisciplinary thinking, as well as mutual learning and otherwise unattainable insights. Previous ethics programs for large-scale science initiatives, such as the Ethical, Legal and Social Implications (ELSI) Program of the Human Genome Project, have been criticized for failing to integrate the ELSI work effectively with the science [7]. Others, like the US National Nanotechnology Initiative [8] and Genome Canada's GE^3^LS Program [9], have made attempts to improve integration through the mandated formation of multidisciplinary research teams, but the extent to which these mandates have led to meaningful integration of ESC considerations remains unclear.

In designing the ESC Program for the GCGH, our goal was to prevent ESC challenges from becoming problems, where possible, by identifying them much further upstream than when research proposals are typically submitted to IRBs for prospective review, and where prevention was not possible, to help solve them. Working closely with foundation staff responsible for R&D program strategy and funding, in addition to individual scientists funded through the GCGH initiative, was a critical element of this design. Rather than viewing ESC issues simply as interesting by-products of complex science, such upstream integration enabled us to better understand how ESC issues present as specific challenges at numerous points along the projects' critical paths, and how they may be amenable to ethical analysis and various ESC solutions or management strategies.

Establishing the necessary working relationships with researchers and foundation staff took time and effort. And we occasionally had to counter two common misconceptions about our work: first, that ESC issues are primarily theoretical and therefore of limited relevance to the day-to-day work of successfully conducting research; and second, that our role was simply to facilitate the science by clearing ESC “bottlenecks" for the researchers. We addressed these concerns by focusing more of our attention on how R&D program staff experienced ESC challenges and their ability to facilitate ESC solutions. We were initially concerned that our increasing interactions with R&D program staff would compromise our objectivity or contribute to this impression. In fact, this has rarely been an issue, because we have been consistent in articulating the importance and value of our independent perspective, and also because the “upstream" ESC problems tend to be poorly characterized and therefore less polarized than some other more well-worn ESC issues. As well, R&D staff turnover has, in some situations, required us to “reset" these interactions and is one of the challenges that can limit the rate of penetration of the ESC program model.

But despite these challenges, we believe that intensifying our interactions with the R&D program staff in particular was helpful in four main ways: 1. It enabled identification of ESC issues early in the critical path of a particular research initiative; 2. It contributed to a normalizing of the idea of shared “ESC thinking," a process we have tried to engender by regularly engaging program staff in dialogue about emerging challenges and what might count as effective “solutions"; 3. It nurtured a sophisticated expert forum for “pressure-testing" our proposed solutions to ESC challenges to help ensure they were feasible, practical, and viable in the contexts in question; and 4. It helped to ensure that potentially viable solutions could be applied to any subsequent research project within the GCGH program, and beyond, in addition to the immediate value for the particular project involved in the initial consultation.

### 2. Privilege Southern Perspectives

Integration of science and ethics gives prominence to the perspectives of R&D program staff and researchers—scientists, social scientists, and humanists alike—in large-scale research endeavors. In global health research, there are particularly complex ethical, social, and cultural dimensions to challenges that arise in host communities that are beyond the knowledge and experience of strictly foreign ESC teams. To address these challenges adequately and appropriately it is necessary not only to incorporate the perspectives of local ESC experts, but to privilege them. Therefore, it is important that an ESC program seek meaningful contributions from these essential yet often under- or unrepresented perspectives, thereby ensuring that investigators and program staff have a sufficient depth of understanding and appreciation of the social, economic, and political contexts within which the proposed research will be conducted [10]. For an ESC program in global health research, this translates into privileging perspectives from the “global South."

Despite a great deal of rhetoric to the contrary, funding programs and individual research programs and projects aimed at addressing key health problems of LMICs continue to arise disproportionately from elite northern institutions. Although this state of affairs reflects real and relevant economic and institutional differences between high- and LMICs, too little attention is paid to how LMIC perspectives can be more successfully brought to bear in the shaping of the agendas and practices of global health research. It has also been argued that the conditions required to support an effective research ethics “system"—to which we would add more meaningful integration of science and ethics—are themselves intimately tied to countries' level of development [11].

Nonetheless, strong representation of Southern perspectives and expertise in the process of identifying and addressing ESC challenges in global health research is critical for: providing cultural guidance, particularly in situations in which differences in the meaning of various research activities can lead to ethically problematic misunderstandings [12]; leveraging lived experiences to enhance interpretation of issues related to relevant LMIC guidelines and regulations; and more readily and knowledgeably providing navigation through complex social and institutional and regulatory structures in the South as science moves closer to various forms of field testing. With this in mind, we recommend that ESC programs focused on global health engage bioethicists from LMICs as co-investigators, staff members, and post-doctoral fellows.

This is not without its challenges, however. In our experience, which relies heavily on each team member to provide substantive input on specific cases and to contribute to the broader evolution and strategic direction of our program, common challenges related to connecting to team members working in LMICs—i.e., unreliable phone and internet services—have often proved debilitating. Similarly, although the initial design of our program was to base one of our three primary programmatic foci at an institution in the South, those plans were stymied by a number of administrative hurdles that stemmed largely from our Northern institution's limited experience with international partnerships and the lack of readily accessible “off-the-shelf" models to help design and guide the development of these partnerships. The specific challenges faced in meaningfully engaging essential yet underrepresented perspectives in other large-scale research initiatives will depend, of course, on the particulars of the initiatives. Nonetheless, we suggest that due consideration be given to who might/should bring those perspectives, followed by planning and feasibility testing of strategies for engagement prior to implementation.

### 3. Build on Specific Cases to Identify and Propose Solutions to Cross-Cutting Issues

Our experience has taught us to not only focus on discrete ESC issues specific to a particular project or program, but also to look for opportunities to devise potential solutions to challenges that cut across numerous research endeavors. Although such cross-cutting “model solutions" may vary significantly in their impact and ultimate value, they lend themselves well to strategic dissemination and are thus useful for stimulating broader dialogue in the field as well as among leaders and decision makers looking for concrete proposals. One path to identifying cross-cutting issues in need of solutions is to work upstream in the research process, as described above, while another is to start by solving problems at the level of specific project consultations and extrapolate key concepts to facilitate development of broad solutions. Three illustrative examples of this latter approach from our work in global health are described below.

*Promote respect through effective and ethical community engagement:* There are myriad examples of how superficial, awkward, hurried, or otherwise disrespectful forms of engagement with individuals and communities in LMICs have jeopardized or prematurely ended global health research or delivery initiatives [13], [14]. And yet, despite the seemingly obvious significance of community engagement (CE), current research ethics guidelines and regulations have an almost exclusive focus on the individual and provide very little guidance about successful interactions with communities or the underlying rationales for what respectful engagement of communities entails [15]. This point has been reinforced most recently in the recommendations of the U.S. Presidential Commission in its aim to “further develop operational guidelines for the protection and ethical treatment of human subjects through the means of community engagement" [16]. From the outset of our program we have prioritized the importance of community engagement (CE), recognizing that the complex human interactions accompanying the introduction of new global health technologies—from new contraceptives to vaccines to TB treatments—can play a critical role in their impact and sustainability.

Our ability to provide effective integrated consultation on CE in specific research projects stems directly from our own empirical research on CE—funded through the ESC Program—which generated insights about how CE can contribute to respectful conduct in research through in-depth case studies in various research contexts. For example, our study of CE at the National Health Research Centre (NHRC) in northern Ghana revealed how incorporating traditional community entry practices into the centre's approach to CE helped to promote respectful conduct by correcting power imbalances between guest researchers and the host community [17]. As well, our study of the CE strategies employed in a long-standing prospective observational cohort study of the genetic epidemiology of HIV among sex workers in Nairobi improved our understanding of the social power of CE practices by demonstrating how research projects can create entirely new communities [18].

These insights and experiences from our empirical research have helped us to effectively shape a number of cross-cutting solutions related to CE. For example, our consultation to help map out ESC considerations for site selection for a caged field trial of genetically modified mosquitoes (GMM) for the control of dengue virus transmission expanded the scope of site selection criteria to include key regulatory and CE considerations [19]. These expanded criteria have been referenced in draft WHO guidance for GMM trials [20], and a subsequent framework for CE in GMM trials that arose from the same collaboration [21] has been cited by the U.S. Presidential Commission for the Study of Bioethical Issues [16] and singled out as a promising *general* approach in an editorial in *Nature* [22].

#### Fill gaps in regulation, governance, policies and guidelines:

Many ESC challenges in global health arise from situations in which regulations, governance mechanisms, policies, or guidelines in relevant jurisdictions are either nonexistent or not tailored sufficiently to the nuances of particular scientific endeavors. Still other challenges arise when asymmetries among various countries' regulatory schemes complicate the uniform implementation of research or delivery activities within a region. We've attempted to meet these challenges by focusing our efforts on identifying, critically analyzing, and proposing solutions to fill the regulatory, governance, and policy gaps encountered in specific research domains, and then seeking broader application and impact for those solutions where feasible and appropriate. In some instances, the solutions proposed have remained limited to specific projects (e.g., the development of a project-specific oversight mechanism for a project involving stem cell research at Peking University) [23], while others have broader implications (e.g., principles for researchers' obligations to participants in observational studies in LMICs, principles for global health data access) [24], [25].

#### Promote and facilitate responsible partnerships with the private sector:

The private sector has enormous capacities in manufacturing, product development, and supply chain infrastructure that could prove valuable in many global health initiatives. But many private companies have been severely criticized for unethical practices. As a result, there is a widespread distrust of the private sector within many public sector and civil society organizations, which results in missed opportunities to leverage private sector capacities to improve global health R&D and delivery in certain circumstances. Driven by the belief that trust and effective collaboration between public and private sector partners can be achieved with the appropriate oversight, policies, and governance mechanisms, we have developed several model solutions focused explicitly on the goal of improving trust and accountability in public-private partnerships (PPPs) [26]–[28]. Our aim has been to build on experiences with specific PPPs (e.g., in infant nutrition, agricultural development) to reduce a vast and seemingly insurmountable problem into discrete aspects—e.g., identify and/or develop useful mechanisms of accountability, declarations of values, codes of conduct—that can be applied and evaluated in a broad set of real world applications.

A standing challenge for the ESC Program has been balancing our responsiveness to demands for ESC consultation in specific cases with the need to maintain an active program of empirical and conceptual research to help ensure that the insights and lessons learned through our consultations can be applied successfully to improve our understanding of cross-cutting ESC issues. This tension should be anticipated by any new ESC program and addressed as a key aspect of the design and funding structure of the program.

### 4. Improve the Evaluation of Strategies, Activities, and Outcomes

The evaluation of the impact of research ethics review and consultation is grossly underdeveloped [3], [4]. As ESC programs achieve greater integration with scientific program development and conduct, and gain more experience with the development and dissemination of model solutions to ESC challenges, it will become increasingly essential to develop the strategies and means to fairly and thoroughly evaluate the extent to which ESC problem-solving can improve the global health research enterprise. As with many complex programs, however, there are few if any natural or obvious measures of impact or effectiveness. Traditional academic metrics like publications and citations are generally poor indicators of the real impact of global health research on, for example, the health of LMIC populations. Further complicating the assessment of ESC programs' attributable impact on global health is the fact that their greatest successes may be in preventing the undesirable—but not inevitable—from occurring.

Through trial and error, we have come to recognize that meaningful and rigorous evaluation of the impact of the ESC Program requires us to look beyond simple evaluation practices to embrace new methods for the evaluation of complex interventions [29]. For example, over the course of the evolution of the ESC Program we have progressively shifted our focus toward improving our “program theory" of how the ESC Program works; that is, what are its essential components and what pathways link them with specific outcomes? This paper is one product of this type of analysis. One specific insight drawn from complex evaluation has been that our interactions with R&D program staff, described above, create an ongoing context for “co-learning" [29], i.e., opportunities for the ESC Program to gain a better understanding of how ESC challenges arise and how R&D program staff understand and manage them, and opportunities for R&D program staff to contribute to ESC solutions from the outset and scrutinize and critique them during their development. This, in effect, functions as a built-in evaluation mechanism. We continue to develop our evaluation practices and welcome dialogue and collaboration with other groups who are grappling with these same challenges.

## Conclusions

Research ethics permeates the entirety of the modern scientific endeavor: institutions and researchers promote and protect scientific integrity, IRBs protect and promote the interests of human research subjects, and CSREs are increasingly called upon to address ethical issues that can present perplexing obstacles along the critical paths to the responsible realization of scientific and technological advances. In no domain are scientific advances more needed than in global health. We hope, therefore, that in sharing these lessons above we can help ESC programs focused on global health to evolve, improve their practices, and gain prominence. Moreover, the importance of integration, of looking for broad applications of narrowly intended solutions, of bringing diverse perspectives to bear on complex ethical challenges, and of rigorous impact evaluation are by no means limited to global health; as such, we hope these lessons may also prove useful for CSREs focused on a wide range of scientific endeavors.

**Key Features of an ESC Program for Global Health Research**

1. **Integrate ESC consultation with the scientific endeavor.**
2. **Privilege Southern perspectives.**
3. **Build on specific cases to identify and propose solutions to cross-cutting issues.**
  - Promote respect through effective and ethical community engagement.
  - Fill gaps in regulation, governance, policies, and guidelines.
  - Promote and facilitate responsible partnerships with the private sector.
4. **Evaluate strategies, activities, and outcomes.**
